# miRNA-Profiling in Ejaculated and Epididymal Pig Spermatozoa and Their Relation to Fertility after Artificial Insemination

**DOI:** 10.3390/biology11020236

**Published:** 2022-02-01

**Authors:** Cristina A. Martinez, Jordi Roca, Manuel Alvarez-Rodriguez, Heriberto Rodriguez-Martinez

**Affiliations:** 1 Department of Biomedical & Clinical Sciences (BKV), BKH/Obstetrics & Gynaecology, Faculty of Medicine and Health Sciences, Linköping University, SE-58185 Linköping, Sweden; cristina.martinez-serrano@liu.se (C.A.M.); manuel.alvarez-rodriguez@liu.se (M.A.-R.); 2 Department of Medicine and Animal Surgery, Faculty of Veterinary Medicine, University of Murcia, 30100 Murcia, Spain; roca@um.es

**Keywords:** spermatozoa, miRNAs, fertility biomarkers, epididymis, ejaculate, pig

## Abstract

**Simple Summary:**

The present study searched for the presence and abundance of porcine spermatozoa small RNA sequences (microRNAs) that have the potential to alter gene expression patterns. Four different sperm sources were compared: spermatozoa from three different sections of the ejaculate and from the caudal epididymis, also classed as spermatozoa from higher (HF) or lower (LF) fertility boars. Sperm miRNAs were compared using high-output small RNA sequencing. We identified five sperm miRNAs not previously reported in pigs. Differences in abundance of four miRNAs known to affect the expression of genes with key roles in fertility were related to boar fertility. These miRNAs could be used as fertility markers in artificial insemination programs.

**Abstract:**

MicroRNAs (miRNAs) are short non-coding RNAs (20–25 nucleotides in length) capable of regulating gene expression by binding -fully or partially- to the 3’-UTR of target messenger RNA (mRNA). To date, several studies have investigated the role of sperm miRNAs in spermatogenesis and their remaining presence toward fertilization and early embryo development. However, little is known about the miRNA cargo in the different sperm sources and their possible implications in boar fertility. Here, we characterized the differential abundance of miRNAs in spermatozoa from the terminal segment of the epididymis and three different fractions of the pig ejaculate (sperm-peak, sperm-rich, and post-sperm rich) comparing breeding boars with higher (HF) and lower (LF) fertility after artificial insemination (AI) using high-output small RNA sequencing. We identified five sperm miRNAs that, to our knowledge, have not been previously reported in pigs (mir-10386, mir-10390, mir-6516, mir-9788-1, and mir-9788-2). Additionally, four miRNAs (mir-1285, mir-92a, mir-34c, mir-30), were differentially expressed among spermatozoa sourced from ejaculate fractions and the cauda epididymis, and also different abundance was found between HF and LF groups in mir-182, mir-1285, mir-191, and mir-96. These miRNAs target genes with key roles in fertility, sperm survival, immune tolerance, or cell cycle regulation, among others. Linking the current findings with the expression of specific sperm proteins would help predict fertility in future AI-sires.

## 1. Introduction

The expression of more than 60% of all genes is estimated to be under the regulation of non-coding small RNAs (ncsRNAs) [1]. Currently, several families of ncsRNAs: microRNAs (miRNAs), short interfering RNAs (siRNAs), piwi-interacting RNAs (piRNAs), and transfer RNA (tRNA) have been identified within somatic cells [2,3,4], with the miRNA family being the best described among the species studied so far. The miRNAs are endogenous ncsRNA molecules of 20–25 nucleotides (nt) in length, capable of altering the translation of their messenger RNA (mRNA) targets [5], to influence protein production [6,7,8] by regulating protein translation during the cell cycle [9]. The abundance of several miRNAs has been described in mammalian spermatozoa (both in epidydimal and ejaculated spermatozoa [10]). Specifically, studies of miRNAs in spermatozoa searched for their role in spermatogenesis and their remaining presence toward fertilization and early embryo development, attempting to disclose if their presence or absence in mature spermatozoa might be related to abnormal development and functioning [11,12] and/or fertility modulation, as found in the murine species where the transfer of sperm miRNAs into the oocyte after fertilization was associated with differential early embryo development [3,13,14,15,16]. Moreover, Dicer1 (an enzyme required for miRNA synthesis) in deficient mice developed morphologically abnormal spermatids, low sperm motility, and low fertility [17]. In the porcine genome, up to 457 mature miRNAs have been identified and annotated to date (miRbase 22, www.mirbase.org (accessed 2 March 2018)), among which, several of these miRNAs have been previously studied for profile and functionality [18,19]. However, much remains to be unveiled regarding the identification and biological functions of miRNAs in pig spermatozoa, [12,20], and of differences in the expression of specific miRNAs among semen samples, with different sperm morphology and/or motility. Furthermore, pig spermatozoa recovered from the epididymis terminal section (EpiTS, e.g., mature spermatozoa in the cauda and the convoluted first portion of the vas deferens) [21] or from the ejaculate, depicted miRNAs with the ability to influence the expression of target genes partially responsible for spermatogenesis, sperm maturation, and zygote development [22,23]. The boar ejaculate consists of three different major fractions: the pre-sperm fraction (pre-SRF, with absence of spermatozoa), the sperm–rich fraction (SRF), and the post sperm-rich fraction (post-SRF). The SRF can be further divided into a sperm-peak fraction (SPF), consisting of the first 10–15 mL of the SRF holding approximately 25% of the total ejaculate sperm numbers, and being rich in epididymal caudal fluid [24], and the remaining SRF, which maintains an elevated sperm concentration and protein secretions from the vesicular and prostate glands. The post-SRF not only presents relatively fewer spermatozoa but also increasing secretions of vesicular, prostate, and bulbourethral glands [25]. In the present study, we report deep sequencing of miRNAs from spermatozoa collected from three fractions of the ejaculate (ejaculated spermatozoa exposed to different secretions of the accessory sexual glands, namely SPF, rest of SRF and post-SRF) of mature breeding boars. Furthermore, since miRNAs have been suggested as potential biomarkers in breeding programs [26], the boars used in this study were classified as having higher (HF) or lesser (LF) fertility subsequent to commercial artificial insemination (AI) as well as spermatozoa retrieved from their EpiTS. Our aim was the genome-wide identification and profiling of miRNAs in the different sperm sources to disclose which miRNAs were present after sperm maturation and whether they changed after ejaculation. Moreover, since miRNAs are crucial for fertilization and preimplantation embryo development [27,28], we further postulate that although many factors can influence male fertility, the identification of specific miRNAs could help its prognosis. Therefore, we additionally analyzed whether a differential abundance of specific miRNAs between boars with higher- or lower-fertility could have a putative role as fertility biomarkers, by their relation to potential gene targets. Selected preliminary data had been reported elsewhere [29].

## 2. Materials and Methods

### 2.1. Ethics Statement

All experiments were accomplished in accordance with the European Directive 2010/63/EU EEC for animal experiments and accepted by the University of Murcia’s Research Ethics Commission (research code: 639/2012) and the “Regional Committee for Ethical Approval of Animal Experiments” (Linköpings Djurförsöksetiska nämnd) in Linköping, Sweden (permits Dnr 75-12, ID1400 and 03416-2020).

### 2.2. Experimental Design

All boars used in this study (*n* = 6) presented normal semen quality. The miRNA expression profile was studied in in ejaculated spermatozoa manually collected from each of three ejaculate fractions (SPF, rest of SRF, and post-SRF) as well as in epididymal spermatozoa collected from the EpiTS from the same boars, slaughtered due to genetic renewal at the enterprise. The ejaculates (*n* = 18) were monthly collected (one collection/boar/month) in within 3 months. In this same 3-month period, other ejaculates were weekly collected as routine in the AI center and used to prepare commercial insemination doses for farms. In total, 923 sows were inseminated, and after farrowing, both farrowing rate (FR, proportion of inseminated sows that farrowed) and litter size (LS, number of piglets born per farrowing) were recorded. The raw AI-fertility data, provided by Topics Norsvin España (Madrid, Spain), were subjected to statistically correction for factors associated to farm and sow to separate the individual contribution of each boar to fertility; accounted as the deviation of FR (in percentage) and LS (in number) of each boar [29,30]. The boars were then, according to their FR (high-fertility boars: FR > 0.94; low-fertility boars: FR < −0.42) or LS (high-fertility boars: LS > 0.11; low-fertility boars: LS < −0.14), classified as having higher (HF; *n* = 3, 540 AIs) or lower (LF; *n* = 3, 315 AIs) fertility. The miRNAs from HF and LF boar spermatozoa (ejaculated and epididymal) were compared.

### 2.3. Boars Handling and Sample Collection

Spermatozoa from ejaculates and the epididymis terminal section (EpiTS) used in the experiment were collected from six healthy, mature cross-bred boars (Landrace × Large-White breeds; 2–3 years old) of known fertility, housed in a commercial AI-station (Topigs Norsvin España, Calasparra, Murcia, Spain). To ensure good semen production over time, the boars were housed in a building with constant lighting 16 h per day and equipped with evaporative coolers to maintain temperature and air humidity within comfortable ranges.

Using the gloved-hand method, three separate fractions of the ejaculate (SPF, SRF and post-SRF) were collected from each boar over a period of three months (one ejaculation per boar and month). All ejaculates used in the experiment fulfilled the requirements of sperm quantity and quality to produce commercial AI-doses (> 200 × 106 spermatozoa/mL, >70% progressive motile and >75% with normal morphology). Considering the ejaculates were fractioned, the concentration of spermatozoa was measured (SP-100 NucleoCounter; ChemoMetec A/S, Allerød, Denmark) in a recomposed sample (mixing specific aliquots of the fractions) while sperm motility was assessed using CASA system (ISASV1^®^ CASA; Proiser R + D, Valencia, Spain) and sperm morphology assessed with phase contrast microscopy. The results were compared to data of previous and posterior ejaculates, and they were found to be non-deviating. Each of the three ejaculate fractions was double centrifuged (1500× *g* at room temperature (RT) for 10 min (Rotofix 32A; Hettich Centrifuge UK, Newport Pagnell, Buckinghamshire, England, UK), to harvest the spermatozoa. The boars were slaughtered after being culled for genetic improvement decisions while they were still healthy and fertile, to collect the contents of their EpiTS via cannulation [30]. All sperm pellets obtained were kept in 15 or 2 mL-tubes at −80 °C until further analyses.

### 2.4. RNA Extraction

Total RNA was isolated from spermatozoa using a miRNeasy kit (Qiagen, Hilden, Germany) designed to isolate RNA for low quantity samples following the manufacturer’s instructions. Briefly, each sample (200 µL) was thawed on ice and incubated with 200 µL of QIAzol Lysis Reagent (Qiagen, Hilden, Germany) at room temperature (RT) for 2 h. Samples were mixed by pipetting and vortexing, treated with 100 µL of chloroform, hand-shaken (RT, 15 s) and incubated at RT for 3 min. Then, samples were centrifugated (12,000× *g*, 4 °C, 15 min) and the aqueous phase (200 µL) was mixed with 300 µL of 100% ethanol. Samples were placed into a RNeasy MinElute spin column (Qiagen, Hilden, Germany) in a 2 mL collection tube and centrifuged (8000× *g*, RT, 15 s). Several washes were performed to the column (500 µL of RWT buffer (8000× *g*, RT,15 s), 500 µL of RPE buffer (8000× *g*, RT, 15 s), and 500 µL of 80% ethanol (8000× *g*, RT, 2 min), discarding the flow-through after each washing. Subsequently, the column was centrifuged (15,000× *g*, RT, 5 min) and the flow-through was discarded. The RNA was eluted by centrifuging the spin column membrane containing 10 µL nuclease-free water (15,000× *g*, RT, 1 min). Total RNA content and its quality was determined by NanoDrop^®^ 1000 (Thermo Fisher Scientific, Waltham, MA, USA). RNA concentration in the samples ranged from 50 to 100 ng/μL and an A260/A280 ratio of ~1.8 was achieved in all samples, indicating good RNA purity. Samples were kept at −80 °C until further analysis.

### 2.5. Small RNA Library Preparation

Small RNA libraries were built using the Illumina TruSeq Small RNA Sample kit (RS-200-0012 and RS-200-0024: Indexes 1-24, Illumina, San Diego, CA, USA) following the manufacturer’s instructions. The aim of this procedure was to ligate adapters to each end of the RNA molecule, and then reverse-transcribe and -amplify to generate a cDNA library. A gel purification step prepared the library for clustering and sequencing. Briefly, 1 μg total RNA in 5 μL of molecular grade water was used for each sample. The 3’ and 5’ adapters were ligated to the samples by incubation at 28 °C for 1 h in a thermal cycler. Then, samples were subjected to reverse transcription (50 °C for 1 h) followed by amplification by PCR to create cDNA constructs based on the small RNA ligated with 3’ and 5’ adapters. This step selectively enriched RNA fragments with adapter molecules on both ends. Amplification was performed with 2 primers that annealed to the adapter ends. The PCR settings for the amplification step were as follows: 98 °C for 30 s, 14 cycles of 98 °C for 10 s, 60 °C for 30 s, 72 °C for 15 s, and then 72 °C for 10 min and held at 4 °C. Each library was then run on a High Sensitivity DNA chip (Agilent Technologies, Santa Clara, CA, USA). A total of 24 libraries were then pooled in equal molar amounts and run in a 6% TBE gel (Life Technologies, Carlsbad, CA, USA) at 140 V for 55 min. Bands between 140 and 160 bp containing miRNAs were excised from the gels. These gel pieces were centrifuged in a gel breaker tube (IST Engineering, Milpitas, CA, USA) at 20,000× *g* for 2 min to move the gel through the holes of the gel braker tube into the 2 mL tube and incubated on a rotating chamber at RT for 2 h in 200 µL molecular grade water. The cDNA construct was then checked for quantity and quality with the Agilent High Sensitivity DNA Kit (Agilent Technologies, Santa Clara, CA, USA). For clustering, the total of all molarities from the peaks observed in the Bioanalyzer were used and the libraries were normalized to 2 nM using Tris-HCl 10 mM, pH 8.5. The cDNA construct was then denatured and clustered on a single read Illumina V2 flow cell (Illumina, San Diego, CA, USA) and ran on the Illumina NextSeq sequencing platform (NextSeq 500/550; Illumina, San Diego, CA, USA) with a Mid Output kit v2.5, with at least 10 million reads per sample for 150 cycles for with a 6-cycle indexing read.

### 2.6. Overview of Sequencing Performance

A total of 24 small-RNA libraries were performed (Boars *n* = 6 × Sperm sources *n* = 4) (six boars and four sperm sources per boar), which were internally quality filtered using the procedure chastity pass filter (PF, i.e., the ratio of the brightest base intensity divided by the sum of the brightest and second brightest base intensities) (Illumina System Guide (15050091 v03)) that Illumina’s NextSeq sequencer performs to calculate the percent of passing on patterned and non-patterned Illumina flow cells. The NextSeq flow cell contained four physical lanes and the pooled library was loaded in all lanes. The outcomes of the sequencing process are depicted in Table 1 and Supplementary File S1.

### 2.7. Bioinformatic Analyses

Prior to alignment, reads were trimmed for 3’ and 5’ adapters using Trimmomatic (version 0.36) [31]. The ENCODE miRNA-Seq data were processed using STAR aligner v. 2.4.2a [32]. Clean reads were aligned on porcine genome (ssc10.2), and miRNA-Seq data were studied using Partek Genomics Suite 7.0 (Partek). A Principal Component Analysis (PCA) was performed on all samples (Supplementary File S1). Data were first normalized using the total count normalization method [33,34,35,36]. Differential expression of miRNAs among sperm sources was established by using a one-way ANOVA, setting parameters as a fold change (FC) > 1 or < −1 with *p*-value < 0.05. The raw datasets were deposited at Sequence Read Archive (SRA) with the BioProject accession number: PRJNA762225 (https://www.ncbi.nlm.nih.gov/sra/PRJNA762225 accessed on 12 March 2018).

### 2.8. Target Gene Prediction and Functional Analysis

In the present study, miRDB (http://mirdb.org (accessed on 12 March 2018)) was used to predict which potential mRNA targets could the differentially expressed sperm miRNAs relate to. For target prediction analyses, the miRNAs were aligned with their human homologous considering that the miRNA-target database has been established exclusively for human and some model organisms. Only target genes with a score >90 (miRbase) were selected.

The network of biological functions and pathways based on the GO and KEGG databases was investigated using Cytoscape Software v3.0.0 (http://www.cytoscape.org/ (accessed on 12 March 2018)) application ClueGO v2.0.3.

## 3. Results

### 3.1. Identification of miRNAs in Spermatozoa from Cauda Epididymis and Ejaculate Fractions

Table 2 lists the miRNAs identified in boar spermatozoa that showed differences in abundance among sperm sources, highlighting six miRNAs that have not been described before in pig spermatozoa and five miRNAs that were significantly dysregulated (*p* < 0.005).

**Figure 1:**
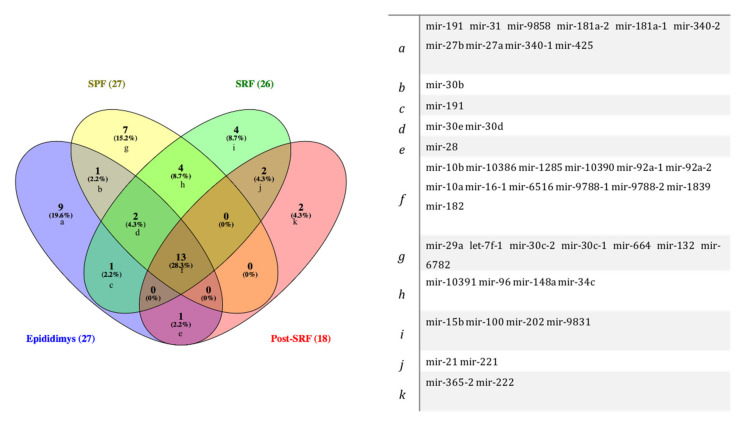
Venn diagram showing microRNAs (miRNAs) commonly identified among spermatozoa recovered from three ejaculate fractions: sperm peak fraction (SPF), sperm rich fraction (SRF), and post-SRF, and from the functional epididymal terminal segment (EpiTS).

Figure 1 depicts the miRNAs commonly expressed among spermatozoa recovered from the three ejaculate fractions studied: SPF (*n* = 27), SRF (*n* = 26), and the post-SRF (*n* = 18), and from the EpiTS (*n* = 27), and where all sources shared the expression of 13 miRNAs.

### 3.2. miRNAs Were Differentially Expressed among Spermatozoa Ejaculate Fractions and from Cauda Epididymis, as Well as between Boars with Higher (HF) or Lower (LF) Fertility: Assessment of Chromosome Location and Structure

**Figure 2:**
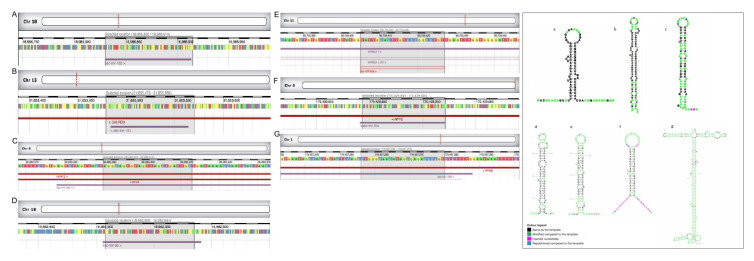
Chromosome location (left image) and miRNA precursor structure (right image) of all differentially expressed microRNAs (miRNAs) (*p*-value < 0.05 and ≥ 1.0-Fold Change (FC) or ≤ −1.0) presented in this study: mir-182 (A,a), mir-191 (B,b), mir-34c-1 (C,c), mir-96 (D,d), mir-92a (E,e), mir-30e (F,f), mir-1285 (G,g). The images were created in Rfam version 14.5 [37]. The black color in the miRNA structure represents a template model, while differences between the template and the sequences are highlighted in color, depending on whether it is a modification (green), an insertion (pink), or a reposition (blue). Structures were generated by R2DT using the d.5.e.P.waltl template provided by CRW [38], copyright © 2021 Rfam Team.

**Figure 3:**
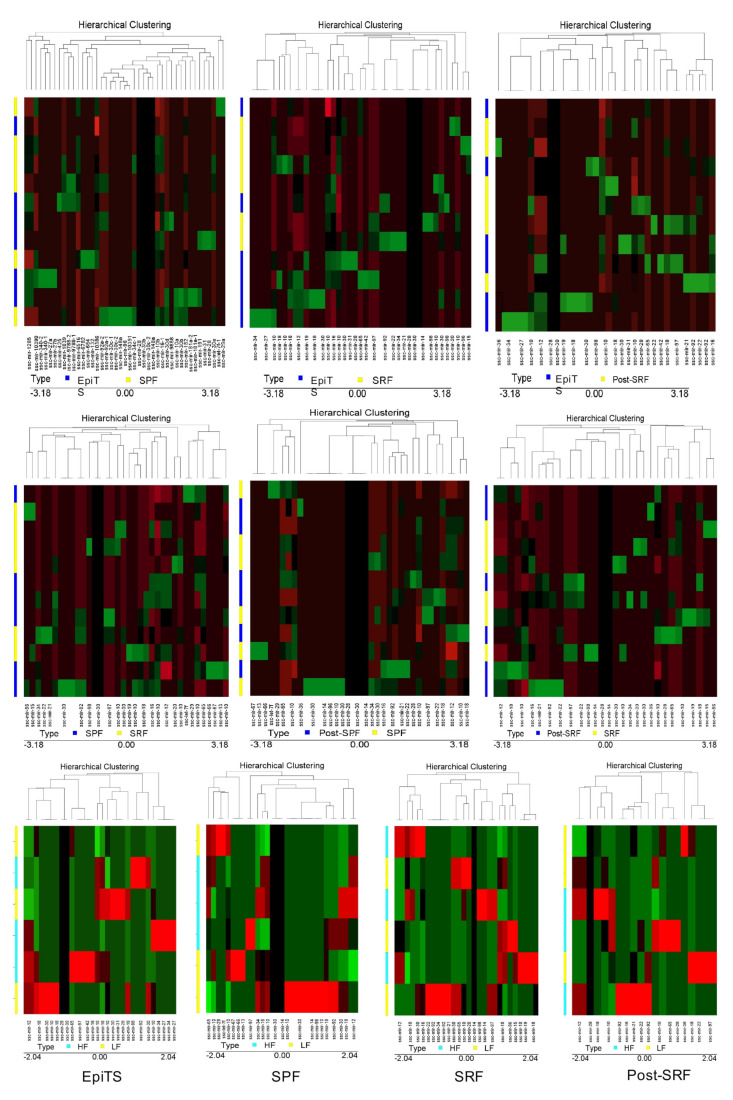
Hierarchical clustering of the expression levels of miRNAs identified in spermatozoa collected from the three ejaculate fractions studied (SPF: sperm-peak fraction; SRF: sperm-rich fraction; and post-SRF) and from the functional epididymal terminal segment (EpiTS) from all boars used in this study (*n* = 6, upper- and mid- figures), and also, expression levels of miRNAs when selecting boars with either higher- (HF) or lower- fertility (LF) (*n* = 3, lower figures). The color scale indicates the relative expression of miRNAs: the red color shows a higher expression and the green color depicts a lower expression. Each row represents one biological sample, and each column represents one miRNA.

Some miRNA abundance differed significantly (*p* < 0.05) among sperm sources (Figure 2). The mir-1285 was upregulated in spermatozoa from the EpiTS compared to spermatozoa derived from any of the ejaculate fractions studied, as well as when comparing the SPF and the post-SRF fractions. The SPF-spermatozoa showed a higher expression of mir-92a-1, mir-92a-2, and mir-34c than spermatozoa from the SRF. The mir-30e was upregulated in SRF-spermatozoa compared with post-SRF spermatozoa (Table 3). Additionally, there was an overexpression of mir-191 and mir-96 (in spermatozoa from EpiTS and SRF, respectively), while a repression of mir-182 and mir-1285 was observed in spermatozoa from the SPF and the SRF, respectively, when comparing spermatozoa from boars of higher- or lesser fertility (Table 3). Figure 2 depicts chromosome location and precursor structure of those miRNAs that were differentially expressed. Figure 3 depicts a hierarchical clustering of the pattern followed by all miRNAs in spermatozoa from different sources within all boars (*n* *= 6*) and in boars with higher- or lower-fertility (*n* *= 3*) within the same sperm source.

### 3.3. Target Prediction and Functional Annotations of Differentially Expressed Sperm miRNAs

**Figure 4:**
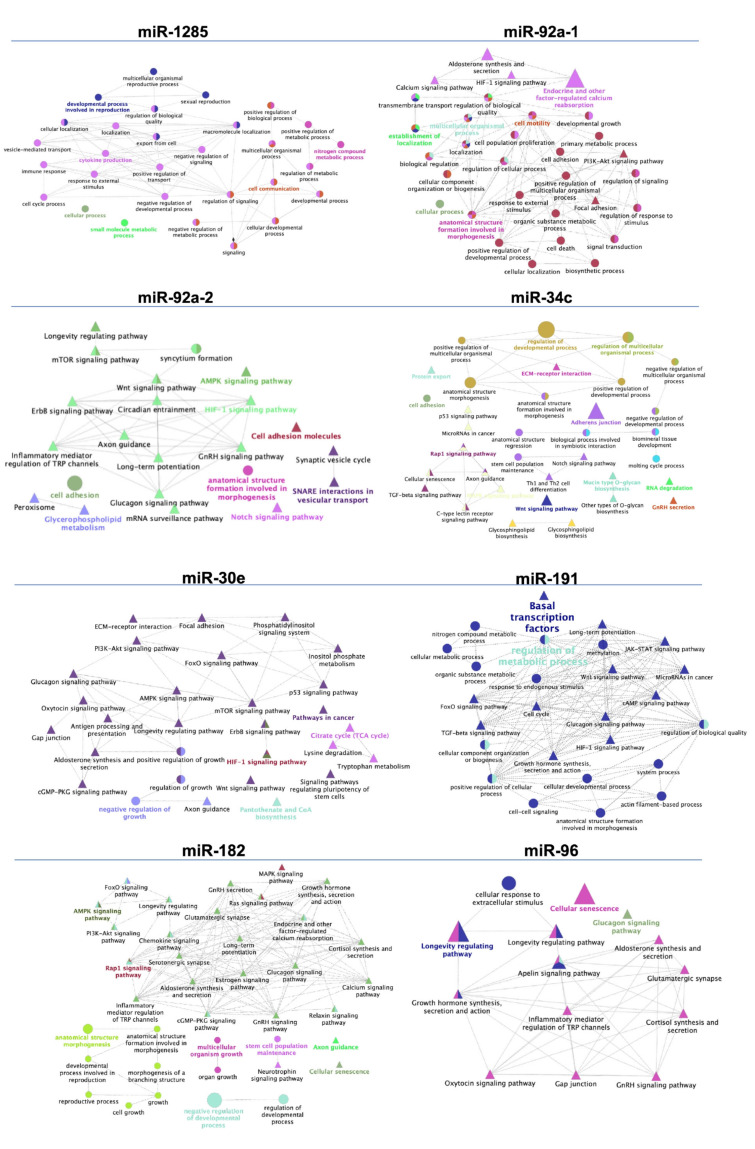
Schematic representation of biological processes and pathways (KEGG) associated with potential target genes for differentially expressed microRNAs (miRNAs, *p*-value < 0.05) found in spermatozoa collected either from the three ejaculate fractions (SPF: sperm-peak fraction; SRF: sperm-rich fraction; and post-SRF), or from the functional epididymal terminal segment (EpiTS) among boars (mir-1285, mir-92a-1, mir-92a-2, mir-34c, and miR-30e) or comparing boars with higher- or lower fertility after AI (mir-191, mir-182, mir-96, and mir-1285). Cytoscape v3.0.0 application ClueGO v2.0.3 was used to build the networks for overrepresented biological processes and pathways. Terms are functionally grouped based on shared genes (kappa score) and are shown in different colors. The following ClueGO parameters were used: GO tree levels, 1–3 (first level = 0), minimum number of genes, 2, minimum percentage of genes, 1, GO term fusion, GO term connection restriction (kappa score), 0.4. Some redundant or unnecessary terms were discarded, and the network was manually rearranged.

To gain insight into the biological function of the differentially expressed sperm miRNAs found in this study, we identified which target genes whose expressions could be potentially and/or partially regulated by these sperm miRNAs. We focused on those target genes with a score ≥90 of homology (Table 4 and Table 5) in the miRNA-target human database (miRDB), against which the miRNAs were aligned owing to the highly conserved degree of homology among species. A total number of 246 potential target genes likely influenced by the expression of the five miRNAs (mir-1285, mir-92a-1, mir-92a-2, mir-34c, and mir-30e) were found after comparing epididymal spermatozoa with those from ejaculate fractions (Table 4). Additionally, a total of 208 target genes were listed as putatively regulated by four miRNAs (mir-191, mir-182, mir-96, and mir-1285) found comparing spermatozoa from higher- with lower-fertile boars (Table 5). The networks representing interactions between those GO terms and biological pathways are shown in Figure 4. ClueGO software (Cytoscape) revealed the considered miRNA-targeted genes (target genes scoring > 90 in miRbase) in several immune-related pathways and cellular processes.

## 4. Discussion

Multiple lines of evidence indicate that sperm miRNAs have crucial gene-regulatory roles in many fundamental biological processes, including spermatogenesis, prompting for sperm motility, fertilization, and early embryo development [14,15,19]. Although pig spermatozoa have been shown to express miRNAs ruling sperm motility, structural integrity and metabolism before [12,39], the present study aimed to perform comprehensive miRNA expression profiling of pig spermatozoa retrieved from different sources using a genome-wide deep sequencing approach. The results have provided novel findings regarding the miRNA content and its differential abundance in the spermatozoa from the EpiTS and from three ejaculate fractions (SPF, SRF, and post-SRF), for their plausible implication in sperm-related biological processes post-fertilization, ultimately influencing fertility by controlling the expression of specific genes.

After sperm production in the testis, spermatozoa acquire motility and fertilizing capacity during their journey along the epididymis [29], interacting with different ncsRNAs (including miRNAs), transcripts, and proteins that are released from the epithelium of the epididymis mostly via epididymosomes [40,41,42]. We have hereby identified a total of 27 miRNAs in spermatozoa from the EpiTS and the SPF, 26 in the SRF, and only 18 in the post-SRF. Interestingly, the differences between the numbers of commonly expressed miRNAs was not large between the SPF and the SRF (*n = 19*) or between EpiTS and SRF (*n = 16*), which seems reasonable considering the SRF (including its SPF) presents higher contents of epidydimal fluid [43].

Some of the miRNAs found in this study have not been described in pig spermatozoa before (mir-10386, mir-10390, mir-10391, mir-9788-1, mir-9788-2, and mir-6516, present in all sperm sources). mir-10386, mir-10390 and mir-10391 have only been described in porcine liver [44]. mir-9788-1 and mir-9788-2 were identified in sow milk exosomes [45], while mir-6516 has been reported in other tissues in several species (porcine liver [44], human platelets [46], chicken embryos, [47] or mouse brain [48]. Further experimental evidence is needed to validate the presence and to identify the roles ascribed to these miRNAs in pig spermatozoa.

Few of the miRNAs identified in the present study were differentially expressed among sperm sources. Mir-1285 was found upregulated in spermatozoa from the epididymis terminal segment compared to spermatozoa from all of the different ejaculate fractions.

This miRNA was previously identified by our group in spermatozoa retrieved from the SRF [49], but, to the best of our knowledge, the present study is the first to report an overexpression of this miRNA in spermatozoa from the EpiTS compared to spermatozoa from the ejaculate fractions. The fact that mir-1285 abundance differed when compared to the other sperm sources could be explained by the fact that, during spermatogenesis, post-transcriptional control of gene expression is highly active [50] and mir-1285 appears to be involved in spermiogenesis by inhibiting boar Sertoli cell proliferation through the regulation of AMPK [51]. Phosphorylated AMPK is implicated in the regulation of 17β-estradiol-mediated inhibition of Sertoli cell viability through increasing p53 and p27 expression and inhibiting mTOR and Skp2 expression [51]. Moreover, mir-1285 regulates the expression of many genes associated with reproductive processes, for instance, the potential target gene *DAZAP1* seems essential for normal development of spermatozoa in mice [52]. This finding supports the theory that miRNAs may play key roles in spermatogenesis. Additionally, we observed an overexpression of three miRNAs (mir-92a-1, mir-92a-2, and mir-34c) in SPF compared with the SRF spermatozoa. The differences in miRNAs abundance between sperm from different parts of the ejaculate suggests that miRNAs regulate mRNA expression in the final processing of spermatozoa during ejaculation [22]. MiR-92 has been shown to play a role in early chicken gonadogenesis by regulating the expression of the *ATRX* and *DDX3X* genes. The mir-92a target genes are implicated in the regulation of the NOTCH signaling pathway, which regulates many cellular events: inhibition of cell differentiation, cell proliferation, and preservation of stem cell population [53]. In addition, NOTCH genes are considered potential targets for mir-34 [54]. The mir-34c belongs to a family of evolutionarily conserved miRNAs (mir-34a, mir-34b, and mir-34c) with active influence on genes that control the cell cycle [55,56], including cell cycle arrest, cellular senescence [57], and apoptosis [58]. Furthermore, the abundance of mir-34c is regulated by the p53 signaling pathway and could constitute a central inhibitor of p53 functions, which are responsible of a variety of intrinsic and extrinsic stress signals that impact upon cellular homeostatic mechanisms regulating DNA replication, chromosome segregation, and cell division [58]. mir-34c has been suggested as fundamental for the development of both male and female bovine gametes and therefore suggested as a potential biomarker of male bovine fertility [59]. Moreover, decreased levels of mir-34c have been observed in samples of infertile individuals [60]. Among the mir-34c target genes, three genes downregulated by mir-34c (*TGIF2, E2F5,* and *BMP3)*, all implicated in the regulation of Transforming Growth Factor-beta (TGF-β) signaling pathway drew our attention. A recent study pointed the TGF-β signaling as a crucial mechanism for immune tolerance to spermatozoa, a very relevant capacity present in the epididymis to tolerate spermatozoa in the lumen, despite they present xenoantigens, which could cause a response from the male immune system [61]. After sperm deposition in the female reproductive tract, similar concepts would apply for the TGF-β signaling pathway, considering its role in preventing spermatozoa from autoimmune responses [49], while simultaneously, the female immune system provides effective protection against ascending pathogens [62]. The genes mentioned above are transcriptional repressors and inhibitors of the TGF-β signaling pathway; its down-regulation triggers the signal elicited by this factor [63,64,65]. The fact that spermatozoa from the SPF may carry epididymosomes containing miRNAs in the cauda epididymal fluid [24,66] could explain these findings. These epididymosomes can provide relevant miRNAs to the spermatozoa besides the endogenous ones above listed, potentially enhancing sperm tolerance and female immune tolerance for a successful fertilization and embryo development, influencing fertility. Overall, the current findings contribute to an increased information regarding sperm miRNAs abundance and possible roles of miRNAs via mRNA regulation on the physiological functions and regulatory mechanisms in pig spermatozoa.

Since the breeding boars used in our study were highly selected for sperm quality, an important requisite for incorporation in AI centers, their fertility was far from being a binary variable, and thus it was defined as of higher- or lower- nature, considering farrowing rate (FR) and litter size (LS) as end-points. Consequently, we additionally investigated whether sperm miRNA expression could be somehow related to boar fertility. Increased abundance of mir-182 (SPF) and mir-1285 (post-SRF) and decreased levels of mir-191 (EpiTS) and mir-96 (SRF) were observed in spermatozoa from higher-fertility boars compared with those retrieved from boars with lower fertility. Moreover, since sperm miRNAs could regulate the expression of genes playing important roles in fertilization and embryo developmental ability, as it has been reported before [14], we further investigated the biological implications of the sperm miRNAs we localized and their target genes.

Mir-191 is improperly expressed in some diseases, including cancer, type 2 diabetes, Crohn’s disease, pulmonary hypertension, and Alzheimer’s disease. Nevertheless, little information has been found regarding its role in reproductive processes. mir-191 was proposed as a key factor involved in embryo development. Additionally, higher concentrations of mir-191 in IVF/ICSI medium were observed in non-successful procedures [67]. Interestingly, mir-191 was found within the epididymosomal miRNA content in the bull [40], suggesting to modulate intercellular communication within the epididymis in low-fertility boars, disrupting epididymal function.

The SPF spermatozoa from boars ranked with higher fertility revealed increased levels of mir-182. This finding adds to the findings of Curry et al., 2011, where high expression of mir-182 was observed in boars depicting high sperm motility and intact structure [39]. Many of the identified miRNA target genes are involved in several cellular processes, including cell structure and growth, cellular senescence and calcium signaling, among others. Of particular interest was the influence of mir-182 on the regulation of *BCL2L12, IGF1R, MED1,* and *RARG* genes, since these genes are implicated in the promotion of cell progression and the negative regulation of apoptotic processes, mainly by direct neutralization of caspase-7 (CASP7) and indirect neutralization of caspase-3 (CASP3), which are known to play essential roles in the execution phase of apoptosis, through the phosphoinositide-3 kinase (PI-3K)-Akt and Ras-Raf-MAPK pathways [68,69]. From our specific analysis of differentially expressed genes, we found an overexpression of three target genes for mir-182 (*ABHD13, MFAP3,* and *PCNX*) in HF compared to LF in the SPF. Of special interest was the *PCNX* gene, which has been speculated to play an important role in the testis, related to spermatogenesis [70]. Overall, these results suggest that mir-182 regulates the survival of spermatozoa towards an adequate progression of spermatozoa within the oviduct. mir-96 has been widely involved in the diagnosis of human cancer progression and development because of its involvement in cellular apoptosis and death [71]. Our results indicate that mir-96 was downregulated in the SRF fraction of the boars depicting higher fertility, but the connection with sperm apoptosis in mature pig spermatozoa is still a debated issue, compared to other species (humans for instance, where the degree of chromatin compaction is lower). In consequence, this issue ought to be followed. *CACNA2D2, SPEN*, and *EBF3* are mir-96 target genes found upregulated in spermatozoa from HF compared to LF boars in the SRF. The calcium channel, voltage-dependent, alpha 2/delta subunit 2 (*CACNA2D2*) regulates calcium current density and is highly expressed in the testis and has been found under expressed in varicocele patients [72]. Mutations of *CACNA2D2* have been associated with reduced male fertility in transgenic mice [73]. *SPEN* (spen family transcription repressor) is a gene that encodes a nucleic acid-binding protein putatively involved in repression of gene expression. *SPEN* is involved in general downregulation of the transcription during the heat shock response in mouse spermatogenic cells through its interactions with chromatin [74], and methylation changes in *EBF3* (Early B cell Factor 3) have been associated with loss of fertility in human [75]. Although the present study revealed some interesting information regarding the abundance of miRNAs in the different sperm sources, it is important to note that other ncsRNAs, such as iRNAs, tsRNAs, or piRNAs are present in spermatozoa, and might be playing an important role in reproductive functions. Further studies are needed to explore potential involvement of other ncsRNAs of interest in sperm function.

## 5. Conclusions

In conclusion, the results presented in this study revealed novel miRNAs in pig spermatozoa whose relative degrees of abundance vary among spermatozoa, from the functional terminal segment of the epididymis and different fractions of the ejaculate. Some of these specific miRNAs could be linked, for homology to predicted target genes with relevant functions related to sperm survival, immune tolerance, or cell cycle regulation, among others. Such regulation could influence embryo development and, ultimately, fertility of the sires, a matter hereby explored. If these genes are essential, then exploration of sperm miRNAs would be beneficial towards the identification of fertility biomarkers, benefiting the efficiency of artificial insemination techniques through safer selections of the most fertile breeders. However, further research is needed to shed evidence on the mechanistic and physiological roles of such miRNAs, and whether they are intrinsic in spermatozoa or derived from epididymosomes and/or seminal plasma.

## Figures and Tables

**Table 1:** Sequencing outcomes for RNA-Seq procedure.

	Total Reads (*n*)	Pf Reads (%)	Reads >q30 (%)	Undetermined Reads (%)
Average Lanes 1–4	14,494,752	97.37	98.39	2.62

**Table 2:** List of identified miRNAs with different abundance among spermatozoa from three ejaculate fractions: sperm peak fraction (SPF), sperm rich fraction (SRF), and post-SRF of mature boars (*n* = 6), and the epididymal terminal segment (EpiTS).

Group	miRNAs (*n*)	Name of miRNAs
**SPF**	27	*mir-182, **mir-6516**, mir-29a, **mir-10386**, mir-let-7f-1, mir-30c-2, mir-30b, mir-96, mir-148a, **mir-92a-1, mir-92a-2**, mir-30d, **mir-10390, mir-9788-1, mir-9788-2**, **mir-1285, mir-34c-1**, mir-30e, mir-30c-1, mir-10a, mir-10391, mir-1839, mir-10b, mir-664, mir-132, mir-6782, mir-16-1*
**SRF**	26	*mir-9831, mir-148a, mir-96, **mir-6516, mir-30e**, mir-15b, mir-21, mir-221, mir-182, **mir-1285**, mir-100, **mir-9788-1, mir-9788-2**, mir-92a-1, mir-34c-1, mir-191, mir-1839, **mir-10391, mir-10386**, **mir-10390**, mir-202, mir-92a-2, mir-10a, mir-30d, mir-16-1, mir-10b*
**Post-SRF**	19	*mir-1285, mir-28, mir-10a, mir-221, **mir-9788-1, mir-9788-2**, mir-182, mir-365-2, **mir-10386**, mir-16-1, mir-1839, **mir-10390, mir-6516**, mir-92a-2, mir-92a-1, mir-10b, mir-21, mir-222, **mir-30e***
**EpiTS**	27	*mir-92a-1, mir-9858, mir-191, mir-27a, mir-340-1, mir-16-1, **mir-10386, mir-1285**, mir-31, mir-92a-2, mir-30d mir-28, mir-30e, **mir-10390**, mir-181a-2, mir-181a-1, mir-10a, mir-10b, mir-30b, mir-340-2, mir-27b, **mir-6516, mir-9788-1, mir-9788-2**, mir-425, mir-1839, mir-182*

miRNAs marked in bold represent not previously described miRNAs in porcine spermatozoa. miRNAs marked in red represent miRNAs with significantly (*p* < 0.05) dysregulated abundance.

**Table 3:** Differentially abundant microRNAs (miRNAs, *p*-value < 0.05) in spermatozoa collected from the three ejaculate fractions studied (SPF: sperm-peak fraction; SRF: sperm-rich fraction; and post-SRF) and from the functional epididymal terminal segment (EpiTS) among all boars or comparing high-and low fertility boars.

Group	Name of miRNAs Differentially Abundant	Fold Change
**SPF vs. SRF**	*mir-34c, mir-92a-1, mir-92a-2*	2.5, 4, 4.1
**SRF vs. Post-SRF**	*mir-30e*	−1.2
**EpiTS vs. SPF**	*mir-1285*	1.7
**EpiTS vs. SRF**	*mir-1285*	1.6
**EpiTS vs. Post-SRF**	*mir-1285*	1.8
**HF vs. LF SPF**	*mir-182*	1.1
**HF vs. LF SRF**	*mir-96*	−3.3
**HF vs. LF Post-SRF**	*mir-1285*	1.1
**HF vs. LF EpiTS**	*mir-191*	−3.1

**Table 4:** List of predicted target genes of differentially expressed miRNAs (*p* value < 0.05 and > 1.0-Fold Change (FC) or< −1.0-FC) in spermatozoa from three different ejaculate fractions: sperm peak fraction (SPF), sperm rich fraction (SRF), or post-SRF, or from the epididymal terminal segment (EpiTS).

Comparison	miRNA	FC	Predicted Targets (*n*)	Name of Predicted Targets
**SPF vs. SRF**	miR-92a-1	4	14	*SLX4 SH3TC2 LMLN NRP2 BCAM PRKCA ATN1 TOB1 ARMC7 ANKS1A SF3A3 ATP2B2 SLC2A1 COL1A1*
	miR-92a-2	4.1	67	*IQSEC2 FOXP4 SYNGAP1 BCL7A FAM222A NFIX TOM1L2 HNF4A NKAIN1 DLK1 STX1B MIER2 ZBTB4 NACC1 APH1A SLC6A17 WNT1 AR CELF5 PPP2R5B RAMP2 KCNQ4 BCAM TGM2 FIBCD1 CELSR2 SLC4A1 NOVA2 ZNF574 PDE1B DNAJB5 ZNF385A ZSWIM4 TFE3 LRCH4 L1CAM MTA1 CLIP2 PPP1R9B GIPC1 EHMT2 B4GALNT1 COL5A3 TSC1 MLLT6 CAMTA1 DUSP3 FAM155B PBX2 SKI SMG5 RIMS3 CAMK2A PAPPA2 C12ORF43 NFIC SLC22A11 HYOU1 SLA2 TNRC6A DMPK SGCD NFASC GNPAT ZNF275 MOCS1 SLIT1*
	miR-34c	2.5	85	*HCN3 FAM76A MDM DLL1 FKBP1B SYT1 E2F5 PPP1R11 RAP1GDS1 FAM167A SDK2 SATB2 SCN2B MYCN NECTIN1 CELF3 MGAT4A LGR4 NAV3 NAV1 MET FLOT2 XYLT1 AHCYL2 TGIF2 PACS1 PKP4 CACNA1E MLLT3 FUT9 RRAS PITPNC1 MPP2 VAMP2 ABR SLC25A27 FOXP1 CAMTA1 SRPRA MEX3C JAKMIP1 ELMOD1 TOB2 FUT8 LEF1 SHANK3 NPNT KIAA1217 GPR22 DAAM1 ASIC2 GALNT7 NUMBL TBL1XR1 BMP3 GABRA3 TNRC18 UBP1 PPARGC1B CUEDC1 ZMYM4 ARID4B FAM117B ATMIN CYREN HNF4A SFT2D1 CDK6 NRN1 EML5 SAR1A TMEM255A FOXN2 TASOR TPPP FGD6 PDE7B ADO ANK3 UNC13C LMAN1 CTNND2 POGZ KDM5D SNAI1*
**SPF vs. Post-SRF**	miR-1285	1.1	18	*SORL1 AASDH ADGRG2 PDE4D PDCD6IP SGCB ANKRD17 SCP2 TWIST2 SNAP25 AFP DAZAP1 ZNF483 C8ORF58 GPBP1L1 ZNF454 PAX5 RFX7*
**SRF vs. Post-SRF**	miR-30e	−1.2	200	*ACTR3C ADAM19 ADAMTS3 ADAMTS9 ADRA2A ALG10 ANKHD1 ANKRA2 ANKRD17 ANO4 ASB3 ATG12 AZIN1 B3GNT5 BDP1 BRD1 BRWD1 BRWD3 C9orf72 CALCR CARF CCDC117 CCDC43 CCDC97 CCNE2 CCNT2 CELSR3 CFL2 CHD1 CHIC1 CHL1 CHST2 CLOCK CNKSR2 CNOT9 COL13A1 COL25A1 CYP24A1 DCTN4 DCUN1D3 DDAH1 DESI2 DLG5 DOLPP1 E2F7 EEA1 EED ELL2 EML1 EML4 EXTL2 FAM160B1 FAP FBXO45 FKBP3 FNDC3A FOXG1 FRMPD1 FRZB FZD3 GABRB1 GALNT7 GMNC GOLGA1 HCFC2 HDAC9 ITGA6 ITPK1 KIAA0408 KLF10 KLF12 KLHL20 KLHL28 LCLAT1 LHX8 LIMCH1 LIN28B LMBR1 LMBR1L LPGAT1 LRRC17 MARCH6 MAST4 MEIOB MEOX2 MIER3 MKRN3 MLXIP MTDH MYH11 NAV3 NCAM1 NECAP1 NEDD4 NEURL1B NFAT5 NFIB NT5E OTUD6B PAPOLA PCDH17 PDE7A PEX5L PFN2 PHIP PHTF2 PIP4K2A PLAGL2 PLEKHM3 PLEKHO2 PLPP6 PLPPR4 PNKD POLR3E PON2 PPARGC1B PPP1R2 PPP3R1 PRDM1 PRLR PRUNE2 PTGFRN PTP4A1 PTPN13 RAP2C RARG RASAL2 REEP3 RFX6 RFX7 RGS8 RIMBP2 ROR1 RORA RRAD RTKN2 RUNX1 RUNX2 S100PBP SAMD8 SCARA5 SCML1 SCN2A SCN3A SCN9A SEC22C SEC23A SEC24A SETD5 SH2B3 SH3PXD2A SIX1 SLC12A6 SLC35A3 SLC35C1 SMAD1 SNX16 SNX18 SOCS1 SOCS3 SOX SPEN SPOCK3 SRSF7 STAC STIM2 STK35 STK39STOX2 STX2 STXBP5 TBC1D10B TBL1XR1 TENT2 TLL2 TMEM170B TMEM181 TMEM56 TNIK TNRC6A TNRC6B TP53INP1 TWF1 UBE2J1 UBE2V2 UBN2 USP37 VIM WDR7 WDR82 XPO1 XPR1 YOD1 YPEL2 YTHDF3 ZBTB11 ZBTB41 ZCCHC2 ZMYND8 ZNRF1*
**EpiTS vs. SPF**	miR-1285	1.7	18	*SORL1 AASDH ADGRG2 PDE4D PDCD6IP SGCB ANKRD17 SCP2 TWIST2 SNAP25 AFP DAZAP1 ZNF483 C8ORF58 GPBP1L1 ZNF454 PAX5 RFX7*
**EpiTS vs. SRF**	miR-1285	1.6	18	*SORL1 AASDH ADGRG2 PDE4D PDCD6IP SGCB ANKRD17 SCP2 TWIST2 SNAP25 AFP DAZAP1 ZNF483 C8ORF58 GPBP1L1 ZNF454 PAX5 RFX7*
**EpiTS vs. Post-SRF**	miR-1285	1.8	18	*SORL1 AASDH ADGRG2 PDE4D PDCD6IP SGCB ANKRD17 SCP2 TWIST2 SNAP25 AFP DAZAP1 ZNF483 C8ORF58 GPBP1L1 ZNF454 PAX5 RFX7*

* Only target genes with a score >90 (miRbase) were selected.

**Table 5:** List of predicted target genes of differentially expressed miRNAs (*p* value < 0.05 and > 1.0-fold change (FC) or < −1.0-FC) in spermatozoa from ejaculate fractions (sperm-peak fraction (SPF), sperm-rich fraction (SRF), and post-SRF), and the functional epididymal terminal segment (EpiTS), and of higher fertility (HF) compared to lower fertility boars (LF).

Comparison	miRNA	Predicted Targets (*n*)	Name of Predicted Targets
**HF- vs. LF-SPF**	miR-182	98	*PRKACB RGS17 BNC2 SNX30 LPP MITF FRS2 CAMSAP2 HAS2 PRRG3 EPAS1 PALLD DCUN1D1 SLC39A9 VAMP3 MTSS1 NPTX1 NEXMIF CD2AP TECTB PRTG SPATA13 SPIN1 CACNB4 MFAP3 CTTN NCALD ACTR2 ABHD13 ADCY6 FOXF2 CADM2 TAF4B EDNRB RAB10 RAPGEF5 LRCH2 CHIC1 ZFP36L1 PCNX1 MAST4 ITPR1 RASA1 LMTK2 USP13 FOXO3 ZC3H15 MAGI1 TAGLN3 RARG LHX1 GNAQ LIMS1 GXYLT1 NRN1 STK19 IGF1R CBFA2T3 FLOT1 HOXA9 BRPF3 CUL5 FAM171A1 MED1 MYRIP TRABD2B PYGO2 PPM1L KIAA1217 HOOK3 SV2C BCL2L12 GIT2 BRMS1L PHIP TMEM47 MIGA1 FNDC3B BNIP3 ZFC3H1 INTS6 DCUN1D3 SLC35G1 PURA PPIL1 SERTAD4 EVI5 ADD3 L1CAM BMT2 STAG1 PLPPR4 ADGRL2 YWHAG HDAC9 ZNF280B RTN4*
**HF- vs. LF-SRF**	miR-96	73	*NEXMIF ADCY6 PRTG SPIN1 FRS2 LRCH2 HAS2 SH3BP5 BRPF3 JMJD1C SNX30 ATXN1 ITPR1 TBR1 PLPPR4 OXSR1 MTSS1 SLC1A1 COL25A1 UBE2G1 B4GALNT4 MED1 PHF20L1 KLHL34 VAMP3 SLAIN2 PHIP RAB8B CTTN E2F5 SOX6 ZFP36L1 SIN3B ZCCHC3 HOOK3 PALLD FOXF2 CHST1 MYRIP ZBTB41 FRMD5 CACNA2D2 PRKCE SH3KBP1 NOVA2 ZEB1 MTOR SLC39A1 PRRG3 TTYH3 NLGN2 FOXO1 ARHGAP6 ANKRD27 SESN3 CEP170B VAT1L PPP4R3A STAG1 CD164 UNC13C DOCK1 SPEN TMEM170B REV1 PPM1L NRN1 MIGA1 STK19 TMEM198 SPAST RGS17 EBF3*
**HF- vs. LF-Post-SRF**	miR-1285	18	*SORL1 AASDH ADGRG2 PDE4D PDCD6IP SGCB ANKRD17 SCP2 TWIST2 SNAP25 AFP DAZAP1 ZNF483 C8ORF58 GPBP1L1 ZNF454 PAX5 RFX7*
**HF- vs. LF-EpiTS**	miR-191	4	*NEURL4 TAF5 CREBB CASTOR2*

Only target genes with a score > 90 (miRbase) were selected.

## Data Availability

The raw datasets generated during and/or analyzed during the current study are available at Sequence Read Archive (SRA) with BioProject accession number: PRJNA762225 (https://www.ncbi.nlm.nih.gov/sra/PRJNA762225 (accessed on 12 March 2018)).

## Supplementary Materials

The following supporting information can be downloaded at: https://www.mdpi.com/article/10.3390/biology11020236/s1, File S1. Sample grouping represented in a PCA plot. Information regarding library quality and sequencing outcomes.
